# The 24-Hour Leukocyte Gap as a novel predictor for sepsis in adult severe blunt trauma

**DOI:** 10.1007/s00423-025-03728-2

**Published:** 2025-06-05

**Authors:** Michel Paul Johan Teuben, Alba Shehu, Ester Mikova, Rald Groven, Christian Huebner, Felix Karl-Ludwig Klingebiel, Roman Pfeifer, Hans-Christoph Pape

**Affiliations:** 1 Department of Trauma, University Hospital Zurich, Zurich, Switzerland; 2 University of Bristol Medical School, University of Bristol, Bristol, UK; 3 Department of Orthopaedic, Trauma, and Reconstructive Surgery, RWTH Aachen University Hospital, Aachen, Germany

**Keywords:** Trauma, Leukocytes, Immunomonitoring, Sepsis, Inflammation, 24 h- leukocyte gap

## Abstract

**Purpose:**

Predicting the likelihood of developing sepsis following severe trauma remains a challenge. As the incidence of sepsis is associated with early post-traumatic episodes of both leukopenia and leukocytosis, various static markers have been trailed in order to help identify and risk stratify patients, nevertheless these have not proven reliable. The goal of this study was to develop and test a novel dynamic immune parameter that could help predict the risk of developing sepsis, the 24-hour leukocyte gap (24 h-LCG), defined as the difference between blood leukocyte numbers on admission and after 24 h.

**Methods:**

A single centre prospective trauma registry was used in order to identify adults who had sustained severe trauma, defined as an Injury Severity Score (ISS) ≥ 9. Patients were stratified into groups based on whether sepsis had occurred. Multivariable regression analysis was performed and utilised in order to analyse predictive immune parameters for sepsis, septic shock and mortality.

**Results:**

1,592 eligible patients were identified, of whom 251 subsequently developed sepsis. Patients diagnosed with sepsis were younger (*p* < 0.002), presented with a higher ISS and had worse hemodynamic parameters on admission (*p* < 0.001). The 24 h-LCG was found to be an independent immunological predictive parameter for sepsis by the multivariable analysis. Moreover, a 24 h-LCG greater than 10, was associated with a significantly increased incidence of septic shock (12.4% vs. 4.3%, *p* < 0.001) and mortality 6.0 vs. 2.7%, *p* = 0.036), compared with the control group.

**Conclusion:**

This study is the first to demonstrate that 24-hour LCG has clinical potential as an independent and early predictive parameter of sepsis in severely injured trauma patients. Furthermore, its feasibility and clinical translatability comes from the use of routine laboratory measurements, namely leukocytes. Its potential lies in assisting future clinical decision making, particularly with regard to the timing of surgery in trauma patients.

## Introduction

Sepsis is one of the most common causes of death in severely injured trauma patients, following traumatic brain injury and early haemorrhage. [1, 2]. Over the past decade, advances in optimising treatment on surgical intensive care units (ICUs) have improved sepsis outcomes in the general population, but not in trauma cases [3, 4]. In the context of trauma, earlier identification of patients at risk of developing sepsis may help to improve patient outcomes and mortality and bring them in line with those seen in the general population. The feasibility of numerous, both conventionally available and expensive, novel (bio)markers to predict the likelihood of developing sepsis upon sustaining a major trauma have been studied before. Nevertheless, early risk stratification of sepsis in a heterogeneous population of trauma patients stills remains suboptimal [5–8]. The role of instant shifts in systemic leukocytes, as indicator of innate cellular immune homeostasis during the early post-traumatic immune response, has not been analysed in a large clinical setting.

Interestingly, an early alteration in systemic leukocyte homeostasis, as reflected by the degree of leukopenia or leukocytosis, have both been linked with worse outcomes in trauma [9, 10]. Furthermore, post-mortem studies of trauma fatalities caused by post-injury inflammatory complications, have shown evidence of massive pulmonary leukocyte infiltration [11]. This highlights the essential role of aberrant neutrophil homeostasis in the development of life-threatening inflammatory complications, such as sepsis, following trauma [12, 13]. However, specific static variables that reflect cellular innate immune homeostasis have been found to be unable to reliably predict the incidence of inflammatory complications [5–8]. Based on the available evidence, a novel dynamic parameter, namely the 24-hour leukocyte gap (24 h-LCG) was proposed and tested for its predictive value. The 24 h-LCG is calculated from serial routine laboratory haematological testing conducted at admission and after 24 h of hospitalisation. The aim of the study was to test the following two hypotheses:

i. *The 24 h-LCG is predictive for septic in-hospital complications in adult blunt trauma patients.*
ii. *A specific subset of patients with an extensive 24 h-LCG is associated with high incidences of septic shock and mortality.*

## Materials and methods

In order to identify eligible patients, the prospective trauma database, maintained by the University Hospital Zurich (a level one trauma centre), was searched. The study was approved and later reapproved by the local institutional review board (registration number: StV 1-2008/PB_2016 − 01888 and BASEC 2021-00391). All procedures in this study were performed in accordance with the ethical standards of the Helsinki Declaration of and its subsequent amendments [14].

**Fig. 1:**
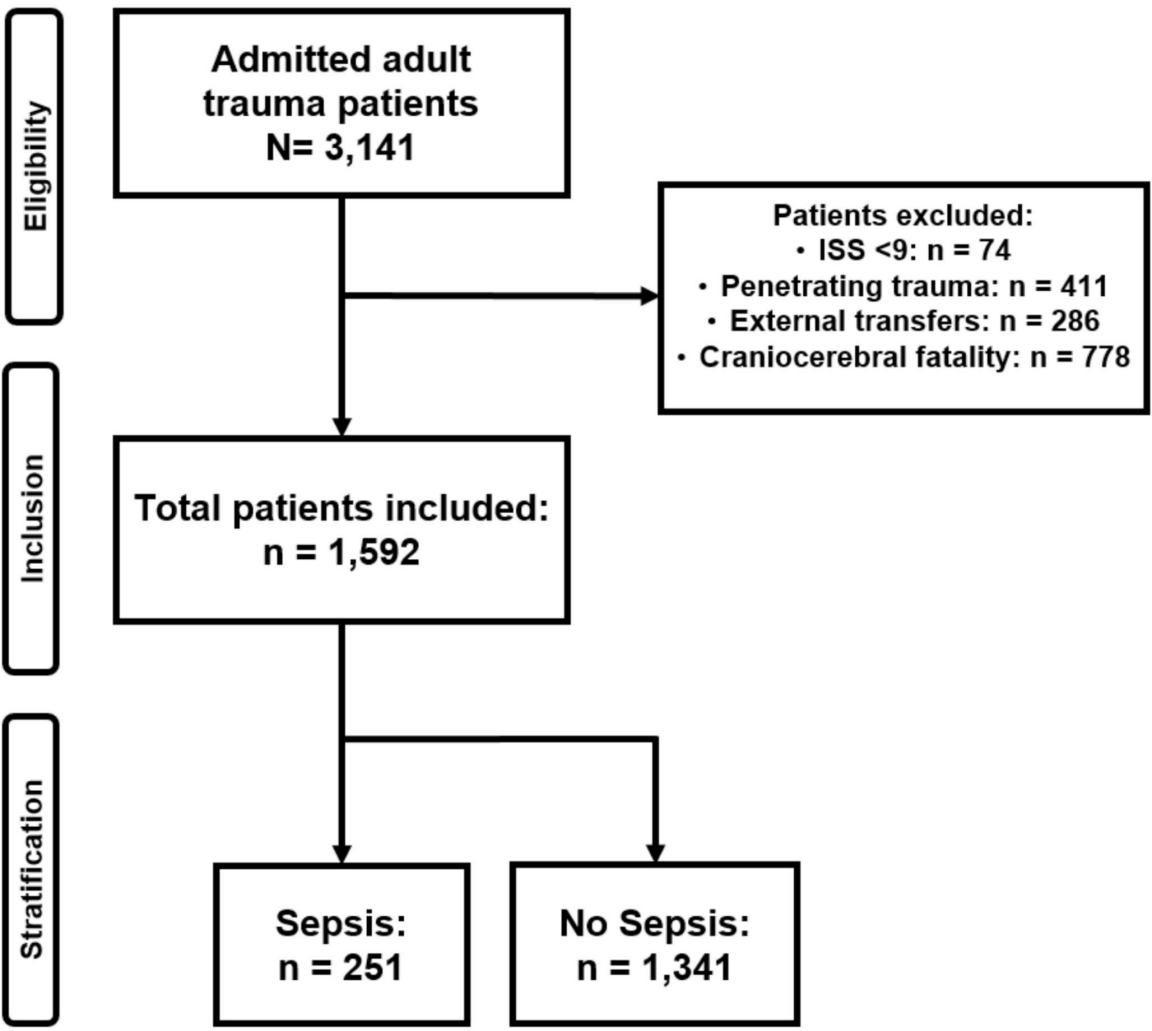
This flowchart displays patient inclusion and exclusion as well as the occurrence of in hospital sepsis. Patient eligibility, inclusion and stratification criteria

All adult patients (aged ≥ 18 years), admitted with severe trauma (defined as an Injury Severity Score (ISS) ≥ 9) [15]) were considered eligible. Patients with penetrating trauma and those with severe craniocerebral injury were excluded from the data set. Additionally, patients transferred from other hospitals or those with missing admission leukocyte counts were not included. See Fig. 1 for patient eligibility, inclusion and stratification.

### Parameters on admission and outcome

To determine baseline patient characteristics, the following data were sourced upon admission: demographics, systolic blood pressure (SBP), pulse rate (PR), Glasgow Coma Scale (GCS), haemoglobin level, lactate level and Injury Severity Score (ISS). Reported outcome parameters included: mortality, incidence of septic shock and sepsis, length of intensive care unit (ICU-LOS) stay, days requiring mechanical ventilation and total hospital stay (HLOS).

Criteria for sepsis and septic shock were defined in accordance with the international standards and the Sepsis-3 criteria. Specifically, septic shock was diagnosed if vasopressor therapy was required to maintain a mean arterial pressure of 65 mm Hg or greater or a serum lactate level greater than 2 mmol/L (> 18 mg/dL) in the absence of hypovolemia [16]. Local protocols and treatment guidelines for trauma care were consistent with international standards [17, 18].

### Static and dynamic laboratory parameters

Both C-reactive protein (CRP) and circulatory leukocyte numbers were measured upon admission and after 24 h of hospitalisation. The first was measured in order to monitor the systemic humoral immune response to trauma, whereas the latter was used in order to assess the trauma-induced cellular immune response in peripheral blood.

In addition to the above-mentioned static laboratory values, a novel parameter was calculated in order to better characterise leukocyte kinetics, and more specifically, to determine the intensity of the immediate circulatory leukocyte response/shift. This parameter, defined as the 24-hour leukocyte gap (24 h-LCG) was calculated as follows:

$$\eqalign{& circulatory\,leukocyte\,numbers\,on\,admission\left( {L{C_{0hr}}} \right) \cr & - circulatory\,leukocyte\,numbers\,after\,24hrs\left( {LC{{24}_{hrs}}} \right) \cr} $$

### Comparison of trauma cases: septis vs. no-septic complications

To test the two hypotheses, several conditions were defined and compared. First, the cases were stratified according to the presence or absence of sepsis, resulting in the following two study-groups:

#### Gr. No-sepsis

no sepsis occurred.

#### Gr. Sepsis

sepsis had been diagnosed.

### Multivariable analysis

A backward stepwise logistic regression analysis was performed to identify which factors predicting the occurrence of sepsis in our population of severely injured trauma patients. First, parameters from the univariable analysis, as described previously, with a *p*-value of less than 0.05 were selected for multivariable analysis. A backward stepwise logit regression was then executed, and the result was further validated by forward regression modelling.

### Subgroup analysis

Finally, based on the regression analysis and upon additional stratification of subgroups, the decision was made to deviate from traditional mediocratic statistical approaches and focus on outliers rather than central bulk values in large data sets with Gaussian distributions. This enabled us to explore the potential of the novel dynamic leukocyte marker, specifically 24 h-LCG, to identify a specific population of trauma patients at high risk for developing sepsis, septic complications and mortality.

### Statistical methods and data processing

All statistical analysis were performed using SPPS (Version 24.0, Chicago, IL, USA). Continuous data was analysed using the T-test or Mann Whitney U Test. The Chi-square/Fisher´s Exact test was used for categorical variables. A significance level of *p* < 0.05 was maintained.

## Results

A total of 1,592 patients were included, of whom 251 were diagnosed with sepsis. Sepsis was diagnosed after a mean/median (IQR) of 9/7 (4–11) days of hospital stay. In 66 individuals, sepsis was diagnosed within the first 72 h after insult. In 74 patients, sepsis occurred during day 4 and 7, in 127 patients, sepsis was diagnosed between day 8 and 14 and later in the remaining 38 patients.

Patients diagnosed with sepsis were significantly younger (41 ± 17 vs. 44 ± 19 years, *p* = 0.002), had a worse cardiopulmonary status (SBP; *p* < 0.001, pulse rate; *p* < 0.001, haemoglobin; *p* < 0.001) and higher ISS (34 ± 13 vs. 26 ± 12, *p* < 0.001) as well as elevated serum lactate levels (3.1 ± 2.3 vs. 2.5 ± 1.6, *p* < 0.001) on admission compared to those individuals who did not develop sepsis. In addition, GCS at admission was significantly lower in patients with sepsis, respectively 7 ± 5 vs. 10 ± 5 (*p* < 0.001). Baseline patient characteristics are displayed in Table 1.

**Table 1:** Baseline patient and trauma characterisitcs

	No sepsis *N* = 1,341	Sepsis *N* = 251	Sign.
Age (years)	44.3 ± 18.6	41.1 ± 17.0	0.002
Gender (%F)	26%	28%	0.501
Systolic Blood Pressure (SBP) (mmHg ± SD)	133.3 ± 24.1	126.8 ± 27.0	< 0.001
Pulse rate (BPM ± SD)	88.7 ± 20.3	94.7 ± 23.9	< 0.001
Glasgow Coma Score (GCS) (± SD)	9.8 ± 5.4	7.0 ± 5.1	< 0.001
Injury Severity Score (ISS) (± SD)	26.2 ± 11.6	33.7 ± 13.0	< 0.001
Lactate (mg/dL ± SD)	2.45 ± 1.61	3.14 ± 2.27	< 0.001
Haemoglobin (g/dL ± SD)	11.8 ± 2.49	11.0 ± 2.89	< 0.001

All data in mean ± SD, Sign based on Mann Whitney-U or T-test

### Differences in kinetics of early circulatory inflammatory parameters between groups

The breakdown of static and dynamic inflammatory markers upon admission and after 24 h are displayed in Table 2. Initial serum CRP levels did not differ significantly between septic and non-septic groups. The circulating leukocyte count at 24 h was significantly lower in patients later diagnosed with sepsis than in those without sepsis (respectively: 8.8 ± 4.2 vs. 9.3 ± 3.6 × 10^9^ cells/L, *p* = 0.016). However, the leukocyte count upon admission did not differ significantly between groups. In addition to the above analysis of static variables, a dynamic parameter, namely the *24 h-Leukocyte Gap (24 h-LCG)*, was calculated. The 24 h-LCG was significantly increased in patients who later developed sepsis (5.2 ± 6.3 vs. 4.0 ± 5.4, *p* < 0.001).

**Table 2:** Static and dynamic inflammatory parameters on admission and after 24 h

	No sepsis	Sepsis	Sign.
*Static parameters*			
CRP on admission (mg/l ± SD)	8,86 ± 29.86	10.87 ± 43.46	0.358
CRP after 24 h (mg/l ± SD)	65.38 ± 56.17	72.56 ± 59.67	0.056
Leukocytes on admission (G/l ± SD)	13.26 ± 5.38	13.94 ± 6.57	0.050
Leukocytes after 24 h (G/l ± SD)	9.31 ± 3.59	8.77 ± 4.15	0.016
*Dynamic parameter*			
Leukocyte gap (24 h-LCG) (± SD)	3.95 ± 5.43	5.17 ± 6.25	0.001

Leukocyte gap = circulatory leukocyte numbers on admission (LC_0hr_)– circulatory leukocyte numbers after 24 h (LC_24hrs_).

#### Higher 24 h-LCG as an independent predictor for sepsis in trauma patients

A stepwise backward logistic regression analysis revealed the following admission parameters: lower GCS/SBP, higher pulse rate and ISS, as well as an increased 24 h-LCG, were all independent predictors of sepsis. Age, admission lactate and haemoglobin levels as well as blood leukocyte counts at day 1 were not significantly predictive of sepsis.

For each 1-point increase in 24 h-LCG, the odds of developing sepsis increased by a factor of 1.03 (3%). Similar findings were demonstrated during the validation process using a stepwise forward logistic regression analysis. The results of the multivariable prediction model are shown in Table 3.

**Table 3:** Results of Stepwise logit regression analysis for the risk of developing sepsis in severely injured patients

	Odds ratio	Lower (95% C.I.)	Upper (95% C.I.)	*P*-value
*GCS-score*	0,941	0,914	0,970	< 0.001
*Systolic blood pressure*	0,993	0,986	0,999	0.022
*Pulse rate*	1,008	1,000	1,015	0.039
*ISS*	1,033	1,021	1,046	< 0.001
24 h-Leukocyte-Gap (24 h-LCG)	1,030	1,003	1,059	0.029
Chi^2^ (df = 5)	**85**,**156**			

Italic values are **static** admission parameters

### 24 h-LCG greater than 10 is associated with highest incidence of sepsis and poorer outcomes

Additional analysis of a specific subset of patients with the most pronounced circulatory leukocyte decline (defined as a 24 h-LCG of ≥ 10, *N* = 251) shows that this specific population has a higher incidence of sepsis compared to regular trauma cases (33.0% vs. 19.5%, *p* < 0.001). Moreover, the duration of ventilator use, ICU-stay and overall hospitalization time were significantly prolonged in this subpopulation of trauma patients (all *p* < 0.001). The incidence of septic shock was approximately three times higher in patients with a 24 h-LCG greater than 10 (12.35% vs. 4.33%, *p* < 0.001) and sepsis/MODS-related mortality was doubled in this population compared with other trauma patients (5.98% vs. 2.68%, *p* < 0.036) (See Table 4). 

**Table 4:** Outcome of trauma patients with a 24-hour leukocyte gap (24 h-LCG) greater than 10

	Regular severe trauma *N* = 1,341	24 h–24 h-LCG ≥ 10 *N* = 251	Sign.
Ventilatory days (± SD)	7.2 ± 9.2	9.6 ± 11.9	< 0.001
ICU stay length (± SD)	11.3 ± 10.9	15.0 ± 15.8	< 0.001
Hospital stay length (days) (± SD)	21.8 ± 17.1	28.1 ± 23.9	< 0.001
Incidence of sepsis	19.5%	33,0%	< 0.001
Incidence of septic shock	4,3%	12,4%	< 0.001
Mortality	10,2%	13,2%	0.181
Sepsis/MODS-related mortality	2,7%	6,0%	0.036

All data in mean ± SD, Sign. based on Mann Whitney-U/T-Tests or Chi-Square-Tests

## Discussion

The main findings of this study can be summarised as follows:

1. In severe trauma, early kinetics of systemic leukocytes better discriminates between patients with and without later septic complications than admission values.
2. Furthermore, this study is the first to show that the a dynamic novel parameter: the 24- hour leukocyte gap (24 h-LCG), defined as the difference between admission and after 24 h circulatory leukocyte number, is an independent predictor for the development sepsis.
3. In addition, a 24 h-LCG > 10 is strongly associated with worse patient outcomes, such as a higher incidence of sepsis, septic shock and sepsis/MODS-related mortality.

These findings imply that in the context of severe trauma in adults, the magnitude of the early immune response largely determines the overall inflammatory response and subsequent clinical course. Additionally, it shows that dynamic analysis of conventional immune parameters ( circulatory leukocytes) may serve as an important predictor of both the early and late immune response which can be linked to the incidence of sepsis and worse patient outcomes. Moreover, leukocyte analysis was found to be superior in comparison to CRP measurements in early discrimination of those trauma patients prone to develop sepsis. 

In contrast to static leukocyte measurements (snapshots such as cell counts or ratios) of immune status following trauma, a dynamic alternative (parameters kinetics) has never been intensively studied before [5–8]. For the purpose of this study, a novel inflammatory parameter was defined and validated: the *24 h-LCG*. This marker reliably reflects the magnitude of the response of the essential effector component of the innate immune response, namely the systemic leukocytes [12, 13], during the first 24 h after severe trauma. The current clinical study implies that a more extensive immune response, reflected by a more profound gap of circulatory leukocyte numbers, tends to occur more frequently in those patients who are prone to develop sepsis.

In line with both experimental and clinical studies, the current study highlights that within the first 24 h since sustaining severe trauma, leukocyte numbers merely tend to drop [9, 19, 20]. In some, but not all cases this leukocyte depletion is preceded by admission leukocytosis [21].

Post-traumatic leukocytosis, as frequently observed in clinical trauma studies, is most likely due to the mobilisation of neutrophils from the marginated pool within the pulmonary circulation and bone marrow [21–23]. Early leukocytosis is triggered by both the initial trauma and later (iatrogenic) insults such as hypothermia, shock, fractures, burns, intubation, mechanical ventilation, hypoxia and invasive interventions/surgeries [13, 24].

Thereafter, and typically still within the first 24 h after injury, circulatory leukocyte numbers tend to decrease in most patients [9, 19, 20]. This is mainly due to neutrophil recruitment into tissue compartments [19, 20]. And it has been demonstrated that excessive leukocyte tissue homing is associated with the development of inflammatory complications such as sepsis [11–13, 25]. Early post-mortem studies have shown that the lungs of trauma victims due to inflammatory complications are characterised by a massive pulmonary presence of inflammatory cells [11]. Furthermore, early post-insult systemic leukocyte depletion occurs due to a mismatch between systemic demands following neutrophil extravasation and instant compensatory neutrophil release from the marginated pool and bone marrow [26–28].

Interestingly, consistent with findings from studies of severe COVID-19 infections, relatively young patients are overrepresented in the groups that develop inflammatory complications after insult (either sepsis or ARDS/organ dysfunction) [29, 30]. It is tempting to speculate that this is not only due to the relatively higher trauma burden in younger patients, but also that the immune system of the young is more at risk of cellular immune dysregulation (over- or underperformance). This issue of age-related changes in immune reactions to trauma should be further investigated in future clinical and experimental studies. 

The 24 h-LCG reflects early systemic leukocyte balancing. The current study is the first of its kind to show that a more profound leukocyte gap is an independent predictor for septic complications. A larger 24 h-LCG reflects a more intense mismatch between systemic demands and compensatory release. Extreme conditions of a systemic leukocyte disbalance, defined as a 24 h-LCG greater than 10 are associated with the highest likelihood of developing sepsis and other complications, such as increased length of hospitalisation or ICU stay.

A large number of blood parameters have been tested for their potential as indicators of the systemic immune response before. Unfortunately, successful implementation of parameters into clinical practice has not occurred yet [5]. Especially in contrast to static parameters, the 24 h-LCG should be considered as a viable alternative to monitor immune response. The 24 h-LCG reflect the response of circulatory cellular immune components within 24 h of sustaining a trauma. A higher value is indicative for a more prominent diversion from leukocyte homeostasis and an increased chance of developing sepsis. Performing serial 24 h-LCG measurements may help to identify the moment when patients become immunologically stable. This may guide the planning of invasive surgical interventions and lead to better overall outcomes.

The specific population of patients with a 24 h-LCG greater than 10 may help explain the paradoxical findings in literature that both leukocytosis and leukopenia are associated worse outcome [9, 19–21, 26]. A high 24 h-LCG has two prerequisites: a state of initial leukocytosis, followed by a state of subsequent leukopenia. It would be interesting to further study this specific patient population in order to develop specific guidelines to best address their needs and optimise outcomes. Within this context serial 24 h-LCG measurements may play a significant role.

This retrospective analysis of a prospectively composed trauma database has several limitations. In order to minimise confounding factors it was decided to work with a complete dataset only. We were not able to determine other inflammatory markers such as interleukin-6 and procalcitonin as these parameters have been added to our routine laboratory trauma panel later on. In addition, the authors chose to exclude patients with severe TBI for two reasons. First, because severe TBI, rather than moderate TBI, affects leukocyte kinetics after injury. Second, it is likely that end-of-life decisions/treatment limitations in the elderly will have a significant impact on outcome analysis, thereby introducing bias [31]. Although, given the importance of TBI and its associated mortality, future studies should focus on outcome prediction and the role of cellular immune responses in this patient population. 

## Conclusion

The current study is the first to describe a cheap and easy-to-use laboratory parameter that can be utilised to monitor early cellular immune response in severe trauma: the 24-hour leukocyte gap (24 h-LCG). The 24 h-LCG is an independent predictor for sepsis in the context of severe trauma in adults. There is potential for the 24 h-LCG to help guide the optimal timing of surgery in the early stages after insult and should be focus of further research. This study highlights the fact that extensive early cellular immune responses, reflected by a 24 h-LCG greater than 10, is associated with poorer outcomes, specifically a three times higher incidence of septic shock and twice as high incidences of sepsis/MODS-associated mortality. These findings could help form the basis of tailored guidelines for this specific subgroup of trauma patients with profound disturbance of homeostasis of the innate cellular immune system, helping to optimise treatment and improve outcome.

## Data Availability

No datasets were generated or analysed during the current study.
